# Insights Into Dolphins' Immunology: Immuno-Phenotypic Study on Mediterranean and Atlantic Stranded Cetaceans

**DOI:** 10.3389/fimmu.2019.00888

**Published:** 2019-04-24

**Authors:** Cinzia Centelleghe, Laura Da Dalt, Letizia Marsili, Rossella Zanetti, Antonio Fernandez, Manuel Arbelo, Eva Sierra, Massimo Castagnaro, Giovanni Di Guardo, Sandro Mazzariol

**Affiliations:** ^1^Department of Comparative Biomedicine and Food Science, University of Padova, Legnaro, Italy; ^2^Department of Physical Sciences, Earth and Environment, University of Siena, Siena, Italy; ^3^Institute of Animal Health and Food Safety, Universitad de Las Palmas de Gran Canaria, Las Palmas, Spain; ^4^Faculty of Veterinary Medicine, University of Teramo, Teramo, Italy

**Keywords:** striped dolphin, bottlenose dolphin, immunohistochemistry, lymphocytes, organochlorine

## Abstract

Immunology of marine mammals is a relatively understudied field and its monitoring plays an important role in the individual and group management of these animals, along with an increasing value as an environmental health indicator. This study was aimed at implementing the knowledge on the immune response in cetaceans stranded along the Italian coastline to provide a baseline useful for assessing the immune status of bottlenose (*Tursiops truncatus*) and striped (*Stenella coeruleoalba*) dolphins. In particular, since the Mediterranean Sea is considered a heavily polluted basin, a comparison with animals living in open waters such as the Atlantic Ocean was made. Formalin-fixed, paraffin-embedded spleen, thymus, and lymph node tissues from 16 animals stranded along Italian and 11 cetaceans from the Canary Island shores were sampled within 48 h from death. Information regarding stranding sites, gender, and age as well as virologic, microbiological, and parasitological investigations, and the cause and/or the death mechanism were also collected in order to carry out statistical analyses. Selected tissues were routinely stained with hematoxylin-eosin (H&E) and with immunohistochemical techniques (IHC). For IHC analysis, anti-human CD5 monoclonal mouse antibody to identify T lymphocytes, CD20 monoclonal mouse antibody for the identification of mature B lymphocytes and HLA-DR antigen (alpha-chain) monoclonal mouse antibody for the identification of the major histocompatibility complex type II were previously validated for both species by Western-blotting technique. *T*-test method applied to quantitative evaluation of IHC positive cells showed a significant relationship between the number of (expression) of CD20 stained lymphocytes and normal and hypoplastic lymph nodes, respectively. No other significant correlations were noticed. Analyses for organochlorines (OC) compounds were performed in animals (n°5) having frozen blubber tissue available. A simple linear regression was calculated to predict if the amount of OCs could influence the number of inflammatory cell subpopulations and a moderate negative correlation was found between the presence of high quantity of contaminants and the number of T lymphocytes. Future analysis should be aimed to understand the effect of the major immunomodulatory pathogens on sub-populations of B and T cells.

## Introduction

Investigating the lymphoid tissues and, more in general, the immunology, and immunopathology of whales and dolphins is relevant to understand the delicate host-pathogen interactions in some of the most relevant infections of cetaceans. For example, Cetacean Morbillivirus (CeMV) enters the immune system and induces an extensive lymphocytolysis causing a generalized lymphoid cell depletion in lymphoid organs. The subsequent immune impairment can then result in an increased susceptibility to secondary infections (1). Beside the direct action of this viral pathogen, a contaminant-induced immunosuppression as a trigger for infectious disease susceptibility's enhancement has been frequently advocated (2–5), highlighting the possible hazards associated with exposure to environmental contaminants, in particular persistent organochlorine (OC) pollutants bioaccumulating in these top predators (6, 7).

Despite the relevance of these studies, descriptions regarding relevant features of the lymphoid system are scanty, fragmented and generally dated, with the first record of lymphoid organs' microscopic anatomy in selected species by Simpson and Gardner (8). Notable exceptions include belugas (*Delphinapterus leucas*) and bottlenose dolphins (*Tursiops truncatus*), in which the morphological architecture of lymphoid organs was extensively investigated (9, 10). Furthermore, an evaluation of cellular and humoral immune responses was carried out again in belugas and harbor porpoises (*Phocoena phocoena*) with an immunophenotyping of their lymphoid cells by an immunohistochemical evaluation of cluster of differentiation (CD) antigens (11).

The specificity of cross-reacting bovine, human, ovine, and murine monoclonal antibodies raised against different leukocyte subsets and the major histocompatibility complex class (MHC) class II antigens of peripheral blood lymphocytes of beluga whales and bottlenose dolphins has been confirmed by immunoprecipitation and flow cytometry (9, 12, 13). Furthermore, cross-reacting antibodies directed against various cell surface antigens of the hematopoietic system, including T cell, B cell, histiocytic, and MHC II antigens have been established for common dolphin (*Delphinus delphis*), striped dolphin (*Stenella coeruleoalba*), bottlenose dolphin, and harbor porpoise lymphoid tissues using immunohistochemistry (14, 15). Similarly, histiocytic cells, particularly resident and inflammatory macrophages can be detected by cross-reacting human antibodies raised against the macrophage-associated antigens CD163, CD204, and lysozyme in short-finned pilot whales (*Globicephala macrorhynchus*) and Risso's dolphins (*Grampus griseus*) using immunohistochemistry (16, 17). Bottlenose dolphin specific monoclonal antibodies for the detection of CD2, CD19, CD21, and CD45R antigens as well as the adhesion molecule beta-2-integrin have been produced and characterized by flow cytometry and immunoprecipitation (13, 18). Furthermore, T cells are recognized by the CD2 marker, while B lymphocytes are predominantly labeled by monoclonal anti-CD19 and -CD21 specific antibodies, using immunohistochemistry (13). Still notably, B cells and a subset of T cells are labeled through the CD45R biomarker (19).

The present investigation was aimed at increasing our knowledge on the immune system of cetaceans, in order to partially fill the existing gaps. Besides characterizing some primary commercial antibodies against cetaceans' leucocytes, this study investigates any possible variations of B and T cells in stranded bottlenose and striped dolphins related both to intrinsic host's factors and to extrinsic insults.

## Materials and Methods

### Animals and Sampling

Formalin-fixed and paraffin embedded lymphoid tissue samples (spleen, lymph nodes, and/or thymus) coming from 27 cetaceans were collected as follow. Tissues from 12 striped dolphins and 4 bottlenose dolphins stranded along the Italian coastlines, or died in aquaria were retrieved from the Mediterranean Marine Mammals Tissue Bank (www.marinemammals.eu). In order to have a comparison with animal coming from different geographical areas, features, 8 striped dolphins, 2 common dolphins, and 1 Risso's dolphin from Canary Island, which succumbed due to ship strikes and with no other pathological findings and/or molecular evidences of infection, were included in the study as “control specimens.” Available data concerning all selected animals and their respective data are summarized in Table 1.

**Table 1:** Data concerning the 27 cetaceans under study^*^.

**ID**	**Species**	**Sex**	**Age class**	**Stranding place**	**Conserv. code**	**Stranding condition**	**Body condition^§^**	**Ongoing infections**	**Other relevant findings**
145	*Tursiops truncatus*	M	Calf	UHC	2	None	Moderate	None	Mild hemorrhagic omphalitis; sub-acute moderate diffuse interstitial pneumonia; moderate to severe multifocal ulcerative gastritis; mild chronic enteritis and moderate chronic colitis with reactive hyperplasia of the GALT.
167	*Stenella coeruleoalba*	M	Adult	Collesalvetti (LI)	2	Dead	Good	None	Nutritional panniculitis, exacerbated by an abnormal localization of Pholeter spp which has significantly reduced the lumen between the first and second gastric chambers.
170	*Stenella coeruleoalba*	F	Adult	Capalbio (GR)	2	Dead	Thin	None	Mild diffuse pyogranulomatous parasitic pneumonia; mild multifocal granulomatous parasitic gastritis associated with a foreign body; acute moderate diffuse catarral enteritis with parasitic infestation.
196	*Tursiops truncatus*	M	Adult	Cervia (RA)	2	Alive	Thin	Toxoplasma spp.	Multifocal moderate pyogranulomatous bronchopneumonia with pulmonary fibrosis and multifocal mild chronic interstitial pneumonia; multifocal severe chronic hepatitis with foci of necrosis and biliary stasis; chronic severe meningo-encephalo-myelitis.
212	*Stenella coeruleoalba*	F	Adult	Livorno (LI)	2	Dead	Moderate	None	Severe diffuse pyogranulomatous pneumonia; mild diffuse chronic endometritis; mild multifocal chronic interstitial nephritis.
214	*Stenella coeruleoalba*	F	Adult	Porto Garibaldi (FE)	3	Dead	Thin	None	Acute moderate multifocal ulcerative esophagitis and stomatitis; severe chronic multifocal parasitic ulcerative gastritis; acute diffuse severe catarrhal gastro-enteritis.
218	*Stenella coeruleoalba*	M	Adult	Lido di Classe (RA)	2	Alive	Good	None	Multifocal mild granulomatous parasitic pneumonia; multifocal moderate ulcerative parasitic gastritis caused by Anisakis spp.; severe diffuse acute catarrhal enteritis; severe multifocal chronic hepatitis.
221	*Stenella coeruleoalba*	M	Adult	Lido di Volano (FE)	2	Alive	Moderate	None	Severe esophageal and gastric food impaction.
229	*Tursiops truncatus*	M	Calf	UHC	1	None	Moderate	None	Moderate multifocal chronic ulcerative gastritis; sub-acute severe diffuse catarral enteritis; meningeal hemorrhages with cerebral edema.
251	*Stenella coeruleoalba*	M	Juvenile	Giugliano (NA)	2	Dead	Good	Morbillivirus	n.a.
255	*Stenella coeruleoalba*	F	Calf	Civitavecchia (RO)	1	Alive	Moderate	None	Moderate multifocal chronic ulcerative stomatitis; severe disseminated granulomatous pneumonia; severe chronic granulomatous parasitic gastritis with partial obstruction of the gastric lumen; severe acute multifocal enteritis.
262	*Stenella coeruleoalba*	M	Juvenile	Napoli (NA)	2	Dead	Moderate	Morbillivirus	n.a.
267	*Stenella coeruleoalba*	F	Adult	Ortoliuzzo (ME)	2	Dead	Good	None	n.a.
273	*Stenella coeruleoalba*	M	Juvenile	Salerno (SA)	2	Dead	Moderate	None	n.a.
327	*Stenella coeruleoalba*	M	Calf	Brancaleone Marina (RC)	1	Alive	Thin	None	Capture myopathy.
343	*Tursiops truncatus*	M	Calf	UHC	1	None	Good	None	Mild to moderate multifocal chronic bronchitis; diffuse and severe hepatic degeneration with capsular multifocal fibrosis; moderate multifocal chronic interstitial nephritis with segmental membranous glomerulopathy.
CET 131	*Delphinus delphis*	M	Calf	Guía de Isora (Tenerife)	2	Dead	Good	None	Trauma by fishing utensil.
CET 151	*Stenella coeruleoalba*	M	Juvenile	La Graciosa (La Graciosa)	1	Alive	Thin	None	Entanglement.
CET 281	*Stenella coeruleoalba*	F	Adult	Puerto del Carmen (Lanzarote)	2	Dead	Good	None	Trauma due to intra/interspecific interaction.
CET 293	*Stenella coeruleoalba*	M	Adult	Arico (Tenerife)	2	Dead	Good	None	Trauma by fishing utensil.
CET 371	*Stenella coeruleoalba*	F	Adult	Arona (Tenerife)	2	Dead	Good	None	Trauma by fishing utensil.
CET 374	*Stenella coeruleoalba*	M	Adult	Playa Tebeto (Fuerteventura)	2	Dead	Moderate	None	Trauma.
CET 406	*Delphinus delphis*	M	Calf	Santiago (Tenerife)	2	Dead	Moderate	None	Trauma due to intra/interspecific interaction.
CET 483	*Grampus griseus*	M	Adult	Puerto del Rosario (Fuerteventura)	2	Dead	Good	None	Trauma due to intra/interspecific interaction.
CET 606	*Stenella coeruleoalba*	F	Adult	Teguise (Lanzarote)	2	Dead	Good	None	Fishing interaction.
CET 616	*Stenella coeruleoalba*	F	Adult	Mogan (Gran Canaria)	2	Alive	Good	None	Collision with ship.
CET 698	*Stenella coeruleoalba*	F	Adult	Los Giunchos (La Palma)	2	Dead	Moderate	None	Trauma consequent to fishing interaction.

* *M, male; F, female; UHC, under human care; n.a., not available*.

§ *, Body condition were defined according to Joblon et al. (20)*.

Briefly, specimens were selected considering the carcass preservation degree [codes 1 and 2 according to Geraci and Loundsbury (21)], along with anamnestic data including sex, age category (calf, juvenile, adult) estimated on total body length and/or on teeth microscopic examination (21), *post mortem* findings. Microbiological and biomolecular investigations for *Morbillivirus* and *T. gondii* were also performed on all major organs (brain, lungs, liver, spleen, lymph nodes, kidnesys) according to already published methodologies [respectively (22, 23)]. Finally, 5 out of the 16 Italian dolphins were selected for ecotoxicological analyses (due to the relevant economic costs of such analyses).

### Western Blotting Analysis

Bottlenose dolphin and striped dolphin (1 g frozen tissue at −80°C) tissue were homogenized using Potter glass (Vetrotecnica, Italia) in 5 ml of buffer A (10 mM Tris-Base, 150 mM NaCl, 5 mM EDTA, pH 7.2 and cocktail inhibitor—Sigma, Milan, Italy) and centrifuged at 10,000 g for 30 min. The supernatant was then centrifuged at 125,000 g for 1 h (Optima L-90K, Beckman, Italy) and the pellet proteins were dissolved in 0.2 ml of buffer B (10 mM Tris, 150 mM pH 7.2 NaCl). Total protein concentration was determined using BCA Protein Assay Kit (Pierce Biotechnology, USA). The samples were diluted 1:1 in 2x Laemmli sample buffer (Sigma–Aldrich, St. Louis, MO, USA), boiled for 5 min at 95°C and separated by 12% SDS–PAGE in a mini-gel apparatus (Hoefer SE 260, GE Healthcare, UK) under denaturing and reducing conditions in according to Laemmli protocol (24). Homogenate of human tonsil was used as a positive control to test the binding with specific antibodies. Following electrophoresis, gels were blotted (350 V, 1 h, 4°C) onto nitrocellulose membranes (0.45 μm; GE Healthcare, UK) in Laemmli transfer buffer (25 mM TRIS-base, 192 mM Glycine and 20% Methanol, pH 8.3) using a trans blot apparatus (Elettrofor, Rovigo, Italy). Membranes were carefully washed in deionized water and blocked overnight at room temperature with 10% skimmed milk and 0.1% Tween-20 (Sigma–Aldrich, St. Louis, MO, USA). Each single membrane was incubated for 1 h at room temperature using the specific antibody diluted in PBS with 0.1% Tween-20 and 5% skim milk. Membranes were washed three times for 10 min with washing buffer (0.1% Tween-20 in PBS) and then incubated for 1 h at room temperature with a horse radish peroxidase (HRP)-conjugated.

Dilution of anti-CD5 (monoclonal rabbit anti-Human; Biocare Medical, USA), anti-CD20 (monoclonal rabbit anti-Human; Thermo Scientific, UK) and anti-HLA-DR (monoclonal mouse anti-Human HLA-DR antigen, Alpha-Chain; DakoCytomation) antibodies (Abs) were defined after appropriate dilution tests and cross-reaction with the secondary anti-rabbit or anti-mouse antibody horse radish peroxidase-conjugated (GAR-HRP and GAM-HRP; BioRad, USA) (Table 2). Finally, the membranes were washed three times with the same washing buffers, and the antigens were visualized by Immobilon Western Chemiluminescent HRP Substrate (MILLIPORE, Billerica, USA) and exposure to autoradiographic films (GE Healthcare, Amersham, UK). Protein bands in autoradiographic films were scanned using an ImageScanner apparatus (Amersham Biosciences, NJ, USA) and analyzed by the software ImageMaster (Total Lab, Amersham Biosciences, NJ, USA).

**Table 2:** Primary Ab dilutions for Western Blotting analyses^*^.

		**Anti-CD5**	**Anti-CD20**	**Anti-HLA-DR**
	mw	58	33	33
Primary Ab		1:500	1:2000	1:2000
Secondary Ab GAR-HRP		1:50000	1:50000	
Secondary Ab GAM-HRP				1:8000

* *mw, molecular weight; GAR-HRP, anti-rabbit antibody horseradish peroxidase-conjugated; GAM-HRP, anti-mouse antibody horseradish peroxidase-conjugated*.

### Microscopic, Immunohistochemical (IHC), and Semi-quantitative Analyses

All the samples were tested by routine microscopic examination and immunohistochemistry (IHC): pre-scapular and/or mediastinal lymph nodes and/or spleen and/or thymus were fixed in 4% buffered formalin, embedded in paraffin and stained for routine microscopic examination using hematoxylin and eosin. In particular, according to Elmore (25) and Valli et al. (26) a lymph node was considered normal if include all the physiological structures (i.e., capsule, subcapsular sinus, cortex, composed by standard predominately B-cell follicles and germinal centers, T-cell-rich paracortical area, medullary sinuses, medullary cords and hilus). Hyperplastic changes were identified by an increase in number and size of follicles and conversion to secondary follicles, involving the B-cell-rich follicles and/or the T-cell-rich paracortex. Reactive follicles were usually larger than the unstimulated ones and had a paler staining germinal center with large lymphoblasts and increased numbers of apoptotic lymphocytes. On the other site, the lack of secondary follicles in the lymph nodes together with the reduced number of primary follicles and the decreased size of their paracortex were indicative of lymph node hypoplasia.

For IHC analysis, staining was performed using an automatic immunostainer (Ventana Benchmark XT, Roche-Diagnostic), which uses a kit with a secondary antibody and with a horseradish peroxidase (HRP)-conjugated polymer that binds mouse and rabbit primary antibodies (ultraViews Universal DAB, Ventana Medical System). All reagents were dispensed automatically except for the primary antibody, which was dispensed by hand. Then we used the anti-CD5 Ab at a dilution of 1:50 and the anti-CD20 Ab at a dilution of 1:800 both incubated for 13 min at room temperature and the anti-HLA-DR, alfa-chain Ab at a dilution of 1:50 incubated for 32 min at room temperature (Table 3).

**Table 3:** Primary Abs used for IHC analysis.

**Mono/ Polyclonal**	**Antibody name**	**Clone**	**Target cells**	**Antigen localization**
Monoclonal Mouse	Anti-human CD5	4C7	T lymphocytes	Cytoplasmic and cell membrane
Monoclonal Rabbit	Anti-human CD20		B lymphocytes	Cell membrane and cytoplasm
Monoclonal Mouse	Anti-human HLA-DR Alpha-chain	TAL.1B5	Antigen presenting cells	Cell membrane

A semi-quantitative analysis was performed using a slide scanner for digital pathology (D-sight, A. Menarini diagnostic). Each IHC-processed section was scanned and immunolabelled cells were counted by two operators within 10 microscopic fields at high power field (40x objective), considered to be representative of the entire lymphoid tissues under investigation. The count was performed manually using an open source image processing program designed for scientific multidimensional images (ImageJ, LOCI, University of Wisconsin-Madison).

Statistical analyses were performed to find possible correlations between the different immune cell populations and independent variables such as species, gender, age class, regional areas, and the presence of ongoing infections.

For statistical analysis the *T*-test was chosen because of the heterogeneity of the samples and the amount of data. A statistically significant threshold was set at a *p*-value of 0.05; a *p*-value < 0.05 was considered indicative of a strong association.

### Toxicological and Statistical Analysis

Analyses for Hexachlorobenzene (HCB), dichlorodiphenyltrichloroethane compounds (DDTs) and polychlorobiphenyl compounds (PCBs) were performed according to methods recommended by the U.S. Environmental Protection Agency (EPA) 8081/8082 with modifications (27) in animals having frozen blubber tissue available at The Mediterranean Marine Mammal Tissue Bank (ID196, ID214, ID218, ID221, and ID229). Blubber samples were lyophilized, and about 1 g each was extracted with n-hexane for gas chromatography (Merck) in a Soxhlet apparatus for analysis of organochlorine compounds. Each sample was spiked with surrogate compound (2,4,6-trichlorobiphenyls—IUPAC number 30) (28) prior to extraction. This compound was quantified and its recovery calculated. Surrogate recovery was reported with the sample results. The samples were then purified. Decachlorobiphenyl (DCBP—IUPAC number 209) was used as an internal standard, added to each sample extract prior to analysis, and included in the calibration standard, constituted by a mixture of specific compounds (Arochlor 1260, HCB and pp'- and op'-DDT, DDD and DDE). The analytical method used was High Resolution Capillary Gas Chromatography with an Agilent 6890 N and a 63Ni ECD and an SBP-5 bonded phase capillary column (30 m long, 0.2 mm i.d.). The carrier gas was nitrogen with a head pressure of 15.5 psi (splitting ratio 50/1). The scavenger gas was argon/methane (95/5) at 40 ml/min. Oven temperature was 100°C for the first 10 min, after which it was increased to 280°C at 5°C/min. Injector and detector temperatures were 200 and 280°C, respectively. The extracted organic material (EOM%) from freeze-dried samples was calculated in all samples. Capillary gas chromatography revealed op'- and pp'- isomers of DDT and its derivatives DDD and DDE, and about 30 PCB congeners. Total PCBs were quantified as the sum of all congeners. These congeners constituted 80% of the total peak area of PCBs in the samples. Total DDTs was calculated as the sum of op'DDT, pp'DDT, op'DDD, pp'DDD, op'DDE, and pp'DDE. The results were expressed in ng/g lipid weight (ng/g lipid weight).

Linear regression analysis was used to study if there was a relationship between the number of the different cellular populations and organochlorines (OC) amount presents in their tissues.

## Results

The present investigation was carried out on a total of 27 dolphins: 20 striped dolphins, 4 bottlenose dolphins, 2 common dolphins and 1 Risso's dolphin; as far as the gender is concerned, 10 individuals were females (37%) and 17 males (63%). Furthermore, 7 of the animals under study (25.9%) were calves, while 4 (14.8%) juveniles and 16 (59.3%) were adults.

### Western Blotting Analysis

Western blotting analysis performed to verify possible cross-reactions of CD5, CD20, and HLA-DR antigens against the secondary antibody did not show specific binding in correspondence of the antigens molecular weight (images not showed). The expression of the CD5 and CD20 antigens was visible at 58kDa and 35kDa and antigens were less visible in striped dolphin (SC) than in bottlenose dolphin (TT) (Supplementary Figures 1A,B). Anti-HLA-DR Ab reacted to the antigens of SC and TT showing a signal corresponding to proteins with a molecular weight of 33kDa with greater intensity in man (Hu) than in SC and TT. The other visible bands were due to non-specific interactions with proteins with molecular weight >50kDa that did not interfere with the proteins of our interest (Supplementary Figure 1C).

The band intensity difference between Hu and SC or TT was probably due to the different antigen concentration of the sample. In the two dolphin's samples there was only cellular membrane extracted and the antigen was more than in the total cellular human tonsil purified.

### Microscopic, Immunohistochemical (IHC), and Semi-quantitative Analyses

The normal microscopic architecture of the lymph nodes varied depending upon location: the major differences involved the amounts of muscle tissue within the *capsula* and the *trabeculae*. The pre-scapular lymph nodes generally showed very little smooth muscle in this location, while in the visceral ones (mainly in mediastinal lymph nodes) smooth muscle encapsulated the node itself and in addition to extending along *trabeculae*, formed an interlacing network throughout the node.

The cetaceans' spleen showed a white pulp composed by lymphoid nodules located at the arterial terminals, evenly distributed throughout the red pulp. Lymphoid nodules were composed of small to medium-sized lymphocytes.

**Figure 1:**
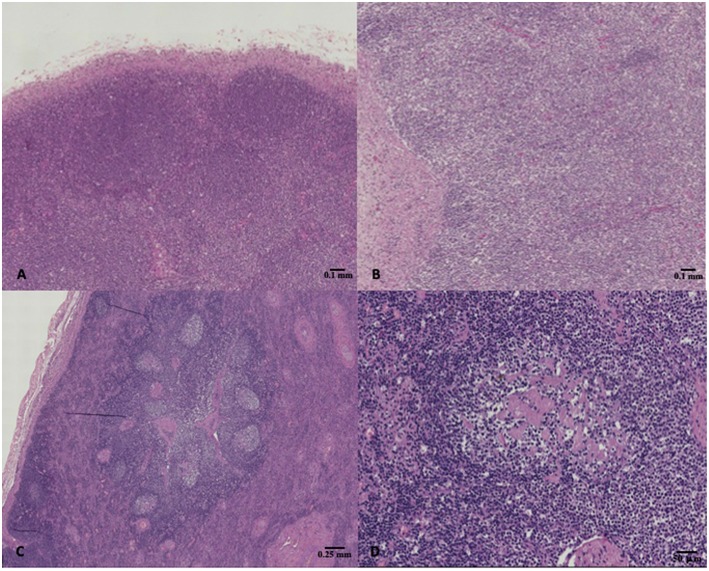
**(A)** Striped dolphin (*Stenella coeruleoalba*) ID 218, normal lymph node, hematoxylin & eosin (H&E), 4X magnification; **(B)** Bottlenose dolphin (*Tursiops truncatus*) ID 229, lymph node hypoplasia characterized by lack of secondary follicles together with the reduced number of primary follicles, H&E, 4X magnification; **(C,D)** Bottlenose dolphin (*Tursiops truncatus*) ID 196, lymph node presenting hyperplastic changes such as reactive follicles which had a paler staining germinal center, H&E, 2X, and 10X magnification respectively.

The results of microscopic examinations carried out on the lymphoid organs of the selected specimens are summarized in Table 4. A total of 11 dolphins showed normal lymphoid tissues, whereas 11 and 5 out of them showed hyperplastic and hypoplastic lymphoid tissues, respectively (Figure 1).

**Table 4:** Results of histological analyses on the lymphoid tissues of the cetaceans under study.

	**Total n^**°**^ of specimen**	**Animal with ongoing diseases**	***Stenella coeruleoalba (20 animal)***	***Tursiops truncates (4 animals)***	**Other species *(3 animals)***	**Mediterranean basin**	**Atlantic basin**
Normal histology	11	0 (11)	9 (11)	1 (11)	1 (11)	6 (11)	4 (11)
Lymphoid hyperplasia	11	1 (11)	6 (11)	3 (11)	2 (11)	3 (11)	6 (11)
Lymphoid hypoplasia	5	2 (5)	4 (5)	1 (5)	0 (5)	4 (5)	1 (5)

**Figure 2:**
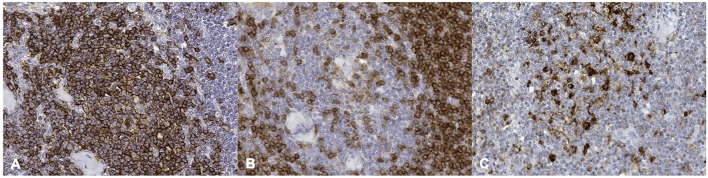
Striped dolphin (*Stenella coeruleoalba*) ID212, lymph node. **(A)** positive cell membrane staining of CD20 antigen; **(B)** positive cell membrane staining of CD5 antigen; **(C)** positive cell membrane staining of HLA-DR antigen (Mayer hematoxylin counterstained; original magnification 40X).

More in detail, positive immunolabeling with the anti-human CD20 Ab was evident within lymphocytes located in the germinal centers, as well as in the mantle and marginal zones of the lymphoid follicles (Figure 2A). As expected, a positive immunostaining reaction against anti-human CD5 Ab was additionally found in lymphocytes located in the paracortical zone (Figure 2B). The medullary area was formed by both CD20 and CD5 positively labeled cells. Cells immunoreactive to anti-human HLA-DR Ab were detected throughout the entire lymph node structure, where antigen-presenting cells were also found to be distributed (Figure 2C).

Normal cetacean thymus follows the typical mammalian organization with a cortex, medulla, and Hassall's corpuscles. The presence of T lymphocytes was demonstrated using the monoclonal anti-human CD5 Ab, with T lymphocytes dominating the entire thymic parenchyma. Few scattered cell clusters lacked MHC class II antigen expression in the thymus.

The average number of B lymphocytes (CD20-immunoreactive, IR), T lymphocytes (CD5-IR) and cells presenting the MHC-II membrane antigen was higher in lymph nodes than in spleen. As anticipated during the IHC qualitative analysis, the thymus did not show CD20-IR lymphocyte. The results of the semi-quantitative analyses on cetacean lymphoid tissues are summarized in Table 5.

**Table 5:** Number of immunoreactive cells in cetacean lymphoid tissues.

	CD5^*^	CD20^*^	HLA-DR^*^
Lymph node	2391.72 ± 1053,96	2398.64 ± 965,97	2158.64 ± 808.91
Spleen	1293.67 ± 877.45	1524.17 ± 336.99	1158.17 ± 438.77
Thymus	2570.67 ± 1694.94	0	3181 ± 1587.98

* *Results expressed as mean ± standard deviation of immunoreactive cells per organ*.

Statistical analysis was performed to evaluate the existence of possible correlations between immune cells populations expression and independent variables such as species, gender, age class, geographic origin/location and the presence of ongoing infections. The only value that approached a strong association (*p*-value < 0.05) was obtained comparing the total number of CD20-IR cells (B lymphocytes) in normal and hypoplastic lymph nodes (0.09). No other statistically significant correlations were found.

No other factor, either intrinsic or extrinsic, appeared to influence the different lymphoid cell marker expression in the specimens under study.

### Toxicological and Statistical Analysis

The blubber organochlorine (OC) levels of IDs 196, 214, 218, 221, and 229 specimens are shown in Table 6. HCB was the contaminant present with the lowest levels in all specimens, with the 3 bottlenose dolphins showing greater concentrations of the all striped dolphins. PCBs were generally higher than DDTs, except for samples of IDs 218 and 221. The main DDT component was pp' DDE. The higher levels of DDTs and PCBs were in one bottlenose dolphin (ID 196), which had about 1124 ppm lipid weight of PCBs and about 430 ppm lipid weight of DDTs, which represent very high levels at an absolute level (29). Very low percentages of extracted organic matter, showing a relevant depletion of blubber layer, indicates a high metabolic stress of the specimens that have mobilized many of your fatty acids, probably due to a sudden weight loss. IDs 214 and 299 had the lowest concentrations of chlorinated xenobiotics, and shows much lower levels compared to other specimens. Despite this, PCB levels of all specimens were greater than the estimated toxicity threshold (17 mg/kg l.w.) set by Jepson et al. (30) and Kannan et al. (31) for cetaceans. This could suggest an important toxicological stress for these cetaceans.

**Table 6:** Results of toxicological analyses.

**Compound**	**ID 196**	**ID 214**	**ID 218**	**ID 221**	**ID 229**
HCB	334.84	89.48	44.95	6n.r.	439.62
30	451.23	266.09	n.r.	56.28	n.r.
95	16588.07	584.08	469.96	799.87	1106.31
op'DDE	1823.45	342.50	441.47	615.93	306.74
101	7267.45	647.43	623.33	1179.92	1062.45
99	113.34	45.86	21.54	39.93	41.13
pp'DDE	306193.29	13268.39	45267.65	52130.65	13803.44
op'DDD	3848.19	243.82	335.49	534.22	315.88
151	23021.64	416.01	622.48	1042.45	650.70
144+135	11355.44	429.08	616.55	967.36	1359.61
149+118	65791.62	1795.42	3112.24	5411.77	3388.50
pp'DDD	11435.87	298.43	981.60	1480.01	965.84
op'DDT	6239.51	309.64	1146.77	1415.65	364.09
146	21296.80	661.01	1212.64	2056.73	1098.32
153	231180.34	3378.12	7414.99	13113.22	4749.11
141	14616.87	206.97	419.90	731.71	219.55
pp'DDT	8387.76	597.06	1249.44	1979.78	658.78
138	124897.00	1743.53	4229.88	7683.19	3391.53
178	12815.13	198.39	419.35	830.33	956.76
187	64919.21	1207.60	2363.71	4366.82	1017.67
183	23533.58	348.56	645.61	1251.99	167.59
128	11117.99	146.62	428.19	764.03	273.17
174	20942.67	335.64	796.84	1544.77	327.74
177	17088.20	224.54	549.64	1025.84	247.06
156 + 171 + 202	11683.37	200.10	424.70	867.35	171.46
172	3338.75	105.79	199.20	467.22	158.07
180	95382.50	1643.69	3222.03	5842.16	631.96
199	619.97	17.61	42.80	46.62	3.82
170	55203.49	786.33	1766.05	3395.17	280.53
196	16114.29	420.94	490.62	1005.19	92.91
201	14128.10	339.57	390.15	719.23	43.03
195	8113.46	267.99	229.00	540.01	38.68
194	9650.27	235.34	260.04	533.63	196.43
206	1191.47	66.28	n.r.	117.87	n.r.
209	418.35	n.r.	n.r.	45.75	n.r.
PCB tot ps	881971.01	16452.51	30971.45	56344.39	21674.07
DDT tot ps	337928.05	15059.84	49422.41	58156.23	16414.76
OC tot ps	1220233.90	31601.83	80438.82	114560.63	38528.46
EOM%	78.50	88.40	51.00	61.80	76.50
HCB bl	426.55	101.23	88.14	97.09	574.67
PCB tot bl	1123529.95	18611.44	60728.34	91172.15	28332.12
DDT tot bl	430481.60	17036.01	96906.69	94103.94	21457.20
OC tot bl	1554438.10	35748.68	157723.17	185373.18	50363.99

**Figure 3:**
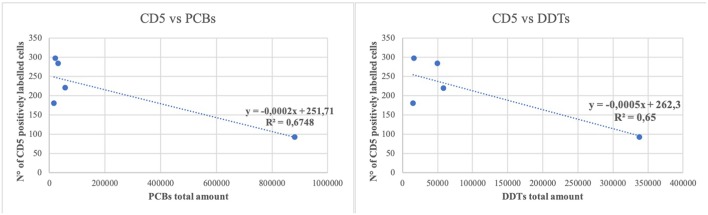
Linear regression between the number of CD5 positively labeled cells and the total amount of polychlorobiphenyl (PCBs) and dichlorodiphenyltrichloroethane compounds (DDTs). Regression lines are reported. R 2 = determination coefficient.

A simple linear regression was calculated to predict if the amount of OCs could influence the number of inflammatory cell subpopulations. A moderate negative correlation was found between the presence of high quantity of contaminants (PCBs and DDTs) and the number of T lymphocytes (*r* = 0.82 and 0.80, respectively) (Figure 3).

## Discussion

The validation of a panel of antibodies reacting against some cetacean antigens opens the possibility of a better understanding of the morpho-functional organization of cetaceans' lymphoid tissues. Although monoclonal antibodies identifying distinct immune cell populations and sub-populations are essential to investigate the role of these cells in the pathogenesis and evolution of different infectious disease conditions, the information about cross-reactivities of antibodies between phylogenetically distant species are important to correctly interpret data obtained from their use (32). Antibodies against human, bovine, mouse and ovine surface antigens such as HLA-DR, CD2, CD4, B cells, and TCR have already been reported to label leukocytes of beluga whale and bottlenose dolphin (9, 13) and different monoclonal antibodies targeting lymphocyte surface antigens for cetaceans were also used in several previous studies aimed at investigating the leukocyte populations residing in cetaceans' lymph nodes (13, 32).

In the present investigation, the antibody specificity validation was achieved following standard guidelines for veterinary laboratories which include, beside the immunoreactivity pattern and the proper use of positive and negative control tissues/matrices, the demonstration of specific immunoreactivity with the respective antigen(s) by Western Blotting analyses (33). The herein investigated Abs resulted valuable for IHC application on formalin-fixed, paraffin-embedded tissues of striped dolphins and bottlenose dolphins, thus mirroring the immunoreactivity patterns observed in other mammalian species (34, 35). Despite the small number of samples, leukocyte subsets' counting and the subsequent statistical analysis revealed that hypoplastic lymphoid tissues were associated with a lower number of B lymphocytes. Interestingly, many animals showing these features (40%) suffered from an ongoing infection, albeit not showing any other morphological alterations.

A possible reason for the decreased B lymphocytes' number could be related to the immune response impairment cause by pathogens like cetacean morbillivirus (CeMV), as already reported in the literature (1). Morbilliviral infections have long been known to result in host immune suppression (36–38), although the most relevant viral effects are a decreased mitogen-induced proliferation and an increase in lysozyme concentration of CD4+ T lymphocytes and a marginally significant increase in monocyte phagocytosis (38).

In this respect, our data seem to be in contrast to those available in the literature, with special reference to CeMV/DMV infection. One of the possible explanation of these apparent discrepancies could be the result of the cetacean host's dominant “immunophenotype.” In this respect, however, despite its pivotal role in driving the evolution and the final outcome of many infectious disease processes, as the natural history of *Human Immunodeficiency Virus* (HIV) infection has taught us (39), we are not aware, thus far, of any study focused on the pathogenetic evolution of CeMV/DMV infection—as well as of *Herpesvirus, Toxoplasma gondii*, and *Brucella ceti* infections—among T helper 1 (Th1)-dominant vs. Th2-dominant cetacean hosts (40).

In addition to infectious agents, lymphoid cell depletion might be caused by environmental contaminants in cetaceans. Although a strong real effect of xenobiotics on the immune system, especially for the dioxin-like poly-chloro-biphenyl compounds (PCBs), has been well established in laboratory rodents (41). Only few studies are focused on the influence of xenobiotics on the immune function of whales and dolphins. In our investigation, preliminary in-depth ecotoxicological analyses carried out on 5 specimens revealed a moderate negative correlation between high tissue levels of PCBs and DDTs and the number of CD3-IR cells (T-lymphocytes). A reduced mitogen-induced T cell proliferation associated with elevated PCB and p,p0-dichlorodiphenyltrichloroethane (DDT) blood levels has been reported in free-ranging bottlenose dolphins on the coast of Florida, suggestive of a contaminant-induced inhibition of the cellular immune response (42). However, definitive conclusions about the impact of the pollutants under study on the health status were limited by the small number of investigated dolphins and by the lack of controls specimens (42). Lymphoid tissue hypoplasia is primarily associated with elevated polybrominated diphenyl ether (PBDE) tissue levels, while there is no apparent correlation with the health and nutritional *status* in bycaught animals, supporting the hypothesis of a contaminant-induced immune deficiency (43). The immunotoxicity of several xenobiotics on cetacean blood leukocytes at concentrations equivalent to those observed in wild marine mammal populations has been also verified *in vitro*. Noteworthy, DDT and non-coplanar PCB congeners inhibited spontaneous and mitogen-induced proliferation of beluga whale lymphoid cells, while coplanar (dioxin-like) PCB congeners and TCDD failed to modulate leukocyte function (19). Further in *vitro* experiments have confirmed the aforementioned inhibitory effects on phagocytosis of neutrophils and monocytes of bottlenose dolphins and beluga whales. The dominating effect of non-coplanar PCB congeners is suggestive of a modulation of the leukocyte function in an aryl hydrocarbon receptor-independent manner in these marine mammals (44). Similarly to T lymphocytes, mitogen-induced proliferation is mainly modulated by POPs and PCBs also in marine mammal B cells (44, 45), although clear-cut effects have not been defined on either lymphocyte populations.

In this respect, the limited number of the investigated cetacean specimens do not allow definitive conclusions. Indeed, non-uniform results were reported also in human beings after chronic exposure to PCBs. For instance, the consumption of pilot whale's (*Globicephala melas*) meat harboring high PCB levels has been associated, in a cohort of children from the Faroe Islands, with a reduced antibody production in response to vaccination against some infectious pathogens as an indirect indicator of the effects on B cell populations (46). On the other hand, children living in highly polluted areas showed a significant increase in B lymphocytes related to chronic PCB exposure, along with a T cell increase and an NK cell decrease, respectively (47).

Recent publications highlighting the re-emerging threats of these substances increase our concerns for marine mammals' health and conservation (7, 48). Future research on wild cetaceans and, more in general, on free-living aquatic mammals should be aimed at evaluating the effect of environmental pollutants on host-pathogen interaction dynamics, thereby assessing whether contaminant-induced immunotoxicity could be related to a CD4+ T helper cells and/or to a CD8+ cytotoxic T cell reduction, together with a decreased splenic humoral immune response, as it happens in laboratory rodents exposed to lipophilic environmental contaminants (11). Further studies are also needed to precisely define the effective role of CeMV/DMV as an immune-suppressive pathogen, with special emphasis on the viral effects on T and B cell populations, along with its interplay with a progressively increasing number of persistent environmental pollutants.

## Ethics Statement

Tissues for this project have been provided by the Mediterranean Marine Mammal Tissue Bank, Department of Comparative Biomedicine and Food Science, University of Padova, and by Institute of Animal Health (University of Las Palmas de Gran Canaria).

## Author Contributions

CC and SM performed Mediterranean dolphins' necropsy, tissue sampling, and contributed to manuscript writing. LD performed western blot analysis, while CC and RZ executed IHC analysis and LM toxicological analysis and all of them contributed to manuscript writing. AF, MA, and ES performed Atlantic dolphins' post-mortem examination and tissue sampling. MC and GDG reviewed critically the manuscript. All authors reviewed and agreed on the current version of the manuscript.

### Conflict of Interest Statement

The authors declare that the research was conducted in the absence of any commercial or financial relationships that could be construed as a potential conflict of interest.

## Supplementary Material

Supplementary Figure 1 Western blot analysis was performed by loading 4 μg of human tonsil (Hu), 2.5 ug of striped dolphin (SC) and bottlenose dolphin (TT) cell membrane extract from lymph node onto an 12% SDS polyacrylamide gel. **(A)** CD5 monoclonal rabbit antibody (Biocare medical, USA product CRM 328) at a dilution of 1:500. **(B)** CD20 polyclonal rabbit antibody (Thermo Scientific product RB-9013) at a dilution of 1:2,000. **(C)** HLA-DR monoclonal mouse antibody (DakoCytomation, Denmark product M0746) at a dilution of 1:2,000.
