# Supplementary figures and images for: Insights Into Dolphins' Immunology: Immuno-Phenotypic Study on Mediterranean and Atlantic Stranded Cetaceans
Source: Front Immunol. 2019 Apr 24;10:888. doi: 10.3389/fimmu.2019.00888 (PMC6499212; doi:10.3389/fimmu.2019.00888)

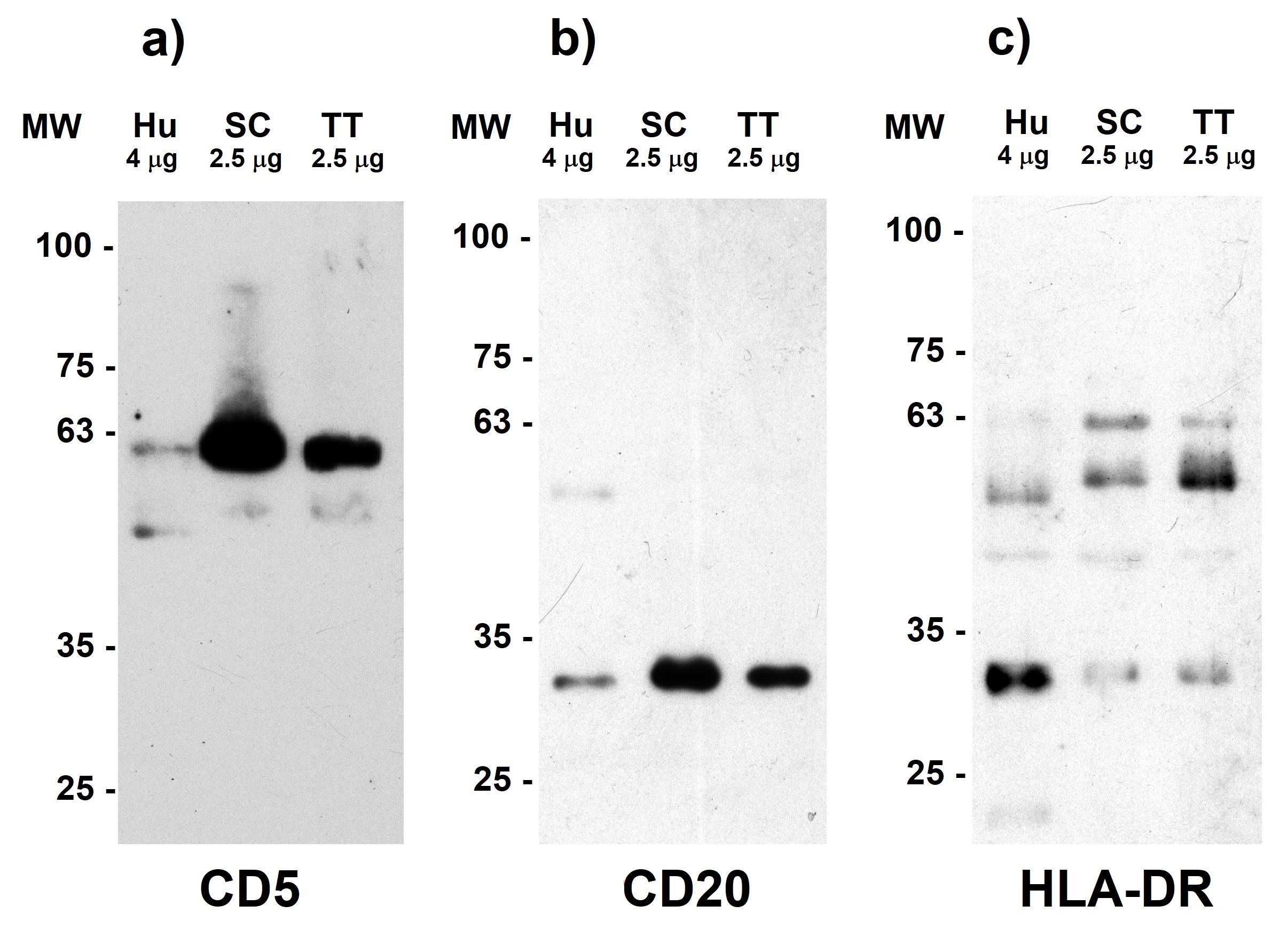

Supplement: Supplementary Figure 1 — Western blot analysis was performed by loading 4 μg of human tonsil (Hu), 2.5 ug of striped dolphin (SC) and bottlenose dolphin (TT) cell membrane extract from lymph node onto an 12% SDS polyacrylamide gel. (A) CD5 monoclonal rabbit antibody (Biocare medical, USA product CRM 328) at a dilution of 1:500. (B) CD20 polyclonal rabbit antibody (Thermo Scientific product RB-9013) at a dilution of 1:2,000. (C) HLA-DR monoclonal mouse antibody (DakoCytomation, Denmark product M0746) at a dilution of 1:2,000. [file Image_1.JPEG]
